# Profiles of patients’ self-reported health after acute stroke

**DOI:** 10.1186/s42466-021-00146-9

**Published:** 2021-08-23

**Authors:** D. Leander Rimmele, Theresa Schrage, Lisa Lebherz, Levente Kriston, Christian Gerloff, Martin Härter, Götz Thomalla

**Affiliations:** 1 Department of Neurology, University Medical Center Hamburg-Eppendorf, Martinistr. 52, 20246 Hamburg, Germany; 2 Department of Medical Psychology, University Medical Center Hamburg-Eppendorf, Martinistr. 52, 20246 Hamburg, Germany

**Keywords:** Stroke, Patient-reported outcome measures, Value-based health care, ICHOM, PROMIS

## Abstract

**Background:**

We aimed to identify groups of patients with similar health status after stroke, assessed by patient reported outcome measures (PROMs), to improve initial risk stratification.

**Methods:**

In a prospective study, inpatients were recruited during acute stroke treatment. Demographics, history, and cardio-vascular risk factors were assessed at baseline. Self-reported functional status, physical and mental health as well as anxiety and depressive symptoms were assessed 3 and 12 months after stroke and used to identify latent classes. The association of patient characteristics with latent class membership was investigated with multinomial logistic regression.

**Results:**

Of the 650 patients included with a mean age of 75 years and 48% female, 70% had ischemic, 6% hemorrhagic strokes, and 24% transient ischemic attacks. Median NIHSS on admission was 2 (IQR:0,5). Values of PROMs remained comparable at 3 and 12 months. A three-class model was developed, differentiating between patients with mildly (75%), moderately (17%), and severely (8%) impaired self-reported health status. Adjusted for univariately significant baseline characteristics, initial NIHSS distinguished mild- from moderate-, and moderate- from severe-class-membership (*p* < 0.001). Length of inpatient stay (*p* < 0.001;OR = 1.1), diabetes (*p* = 0.021;OR = 1.91), and atrial fibrillation (*p* = 0.004;OR = 2.20) predicted allocation to the moderately vs. mildly affected class.

**Conclusions:**

Grading stroke patients by a standard set of PROMs up to 1 year after stroke allows to distinguish the diverse impact of baseline characteristics on differently affected groups. In addition to initial stroke severity, longer inpatient stay, presence of diabetes and atrial fibrillation correlate with greater impairment of self-reported health in the less affected groups.

**Trial registration:**

http://www.ClinicalTrials.gov; Unique identifier: NCT03795948.

**Supplementary Information:**

The online version contains supplementary material available at 10.1186/s42466-021-00146-9.

## Introduction

Prevention and improved treatment of acute stroke has led to a decline in stroke incidence and stroke-related mortality over the past decades. The global burden of stroke, however, remains high due to the prevalence of its chronic stage [14]. Disability caused by stroke may affect a broad range of functions from motor to neuropsychological deficits [7]. With the aim of prevention and intervention, different questionnaires have been developed to characterize and predict the impact of stroke on functional status and quality of life. To this end, a systematic assessment of patient reported outcomes measures (PROMs) has been shown to deliver information beyond routine examinations by physicians and to identify disabling deficits in every-day life after stroke [17, 18]. A possible strategy towards understanding the development of different outcomes and towards risk stratification is classifying patients by the results of their outcomes and analyzing class characteristics, as shown for diabetes [24] and heart transplantation [20]. In mild to moderately affected stroke patients from an outpatient setting, a retrospective analysis showed promising results building distinct profiles or classes using the Patient-reported Outcomes Measurement Information System (PROMIS) [16]. The International Consortium for Health Outcome Measurement Standard Set for Stroke (ICHOM-SSS) has been developed to standardize patient reported outcomes after stroke [30]. We have recently assessed its use in clinical routine at our hospital [27] and amended the Patient-Health-Questionaire-4 (PHQ-4) to address reported depressive symptoms and anxiety after stroke and transient ischemic attacks (TIA). Considering evidence of comparable risk profiles [31] and similar self-reported outcomes in patients with acute stroke and TIA, we decided to assess both, in order to adequately represent the heterogenous stroke unit clientele [4, 16]. We aimed to grade this unselected clientele of patients recruited in a prospective observational study on stroke unit admission and identify latent classes according to health status assessed by PROMS 3 and 12 months after the event.

## Methods

### Study design

The study was designed as a prospective observational analysis for an exploratory longitudinal evaluation of patients with the ICHOM-SSS. Patients were recruited over a 15 month period during initial treatment on the stroke unit of the University Medical Center Hamburg-Eppendorf after diagnosis of acute ischemic stroke (AIS), transient ischemic attack (TIA), intracerebral hemorrhage (ICH), or retinal infarction. Patient-reported outcomes were assessed at follow-up 3 and 12 months after admission. Informed consent was obtained by patients or authorized guardians. The ethics committee of the Hamburg chamber of physicians approved the study protocol (PV54565). The study was registered at ClinicalTrials.gov, NCT03795948, at January 82,019 retrospectively.

### Data acquisition

Data were collected according the ICHOM guidelines and the study protocol [11, 28]. Diagnosis, patient history, demographics, cardio-vascular risk factors, and therapy were collected from interviews and electronic records prior to discharge. The National Institutes of Health Stroke Scale (NIHSS) was examined at admission. Cardiovascular risk factors were defined by presence of relevant diagnoses in medical records or determined by interview. Follow-up assessments of patient-reported outcomes were done at 3 and 12 months after admission by pen-and-paper questionnaire and telephone. They included questions concerning living situation, living location, and recurrent stroke events. Details of assessment have been described previously [27].

### Outcomes

The ICHOM-SSS is based on the PROMIS 10-Question Short Form (PROMIS-10), which contains four items each for mental and physical health used to calculate T-scores. Lower values indicate a poorer outcome. The reference mean is 50 with a standard deviation (SD) of 10 measured in the general population [5].

Functional status post stroke was assessed by one question each for dependence on the help of others for walking (yes, partly, no), dressing (yes, no), and toileting (yes, no), with 0 representing fully independent (high functional status) and 4 fully dependent (low functional status) [11].

We administered the Patient Health Questionnaire-4 (PHQ-4) in addition to the ICHOM-SSS to address symptoms of anxiety and depression. It consists of two items each for potential presence of anxiety and depressive symptoms. Scales for each reach from one to six with values of three and above indicating anxiety or depressive symptoms. Reference mean values of a general population for anxiety are 0.82 (CI:0.79;0.85) and for depressive symptoms 0.94 (CI:0.90;0.97) [21].

### Statistical analysis

All patients with the diagnosis of AIS, TIA, or ICH and at least one valid measurement of PROMIS-10, functional status, or PHQ-4 at 3 or 12 months were included in the analysis. We excluded patients with retinal ischemia from analysis due to different clinical manifestation.

Descriptive and multinomial regression analyses were carried out using IBM SPSS Statistics, Version 25.0 (Armonk, NY: IBM Corp), and JASP Version 0.14. The latent profile analysis was performed in Mplus version 7.2 (Muthén & Muthén, Los Angeles, CA).

### Latent profile analysis

We performed a latent profile analysis (LPA) based on patient-reported health data to identify groups of similar outcome [23]. Patient-reported assessments of physical and mental health, functional status, depressive symptoms, and anxiety 3 and 12 months after acute stroke treatment were used to characterize the groups respectively classes. Class membership was defined by a series of models varying across three dimensions. First, within-class correlations between the analyzed variables were modeled in three different ways: all correlations restricted to be zero, non-zero correlations only between the same variables measured at different timepoints, and non-zero correlations allowed between any of the variables. Second, within-class variance of the same variables measured at different timepoints were either restricted to be equal or allowed to be different. Third, the number of classes were varied from one to six. This results in a total of 3x2x6 = 36 estimated models. We use a robust maximum likelihood estimator with 20,000 starting values for fitting the models. Model selection criteria and fit indices are reported in the supplementary.

### Multinominal regression

After determining the most likely class membership for each patient, we investigated the association of demographic, clinical, and functional parameters with class membership using multinomial logistic regression. The class of mild impairment was opposed to the one of moderate and the class of moderate impairment to severely impaired separately. Binary univariate regression was performed in advance, and parameters associated with class membership with *p* < 0.05 were included in multivariable multinominal logistic regression models. The NIHSS was used in categories of no (0), mild (1–4), moderate (5–15), moderate to severe (16–21) and severe (22–42) symptoms.

## Results

### Patient characteristics and self-reported outcomes

We included 650 patients in the analysis. 70% of them were diagnosed having acute ischemic stroke, 6% intracranial hemorrhage, and 24% transient ischemic attack. Mean age was 75 years, 48% were female, and the median NIHSS assessed on admission was 2.0 (IQR:0,5). 67% of patients lived without need of external support, and 59% lived with a partner. Categorized by the NIHSS, 29.5% had no neurological deficit (NIHSS: 0), 41.8% mild deficits (NIHSS: 1–4), 23.7% moderate deficits (NIHSS: 5–15), 3.4% moderate to severe deficits (NIHSS: 16–20), and 1.5% severe deficits (NIHSS: 21–42). Concerning risk factors, 62% had hypertension, 19% atrial fibrillation, and 15% diabetes.

Their mean reported physical health after 3 and 12 months of follow up measured by PROMIS-10 was 44.7 (SD = 10.00, *n* = 555) and 44.9 (SD = 10.19, *n* = 516). Mean mental health scores were 41.0 (SD = 9.73, *n* = 562) and 41.6 (SD = 10.05, *n* = 522), respectively. Values of physical and mental health were reduced compared to their reference value of 50 (SD = 10) from healthy subjects, with lower values representing increased impairment. Scores of the PHQ-4 after 3 months indicated anxiety in 16.3% (*n* = 90/552; mean = 1.35 ± 1.42) and depressive symptoms in 17.4% (*n* = 96/553; mean = 1.46 ± 1.45) of the patients. After 12 months 16.3% (*n* = 82/502; mean = 1.33 ± 1.50) scored indicative for anxiety and 19.1% (n = 96/503; mean = 1.50 ± 1.53) for depressive symptoms. Functional impairment post-stroke determined by the ICHOM-SSS had a mean value of 0.45 (SD = 1.00, *n* = 568) after three, and of 0.47 (SD = 0.98, *n* = 529) after 12 months.

### Latent classes

**Fig. 1:**
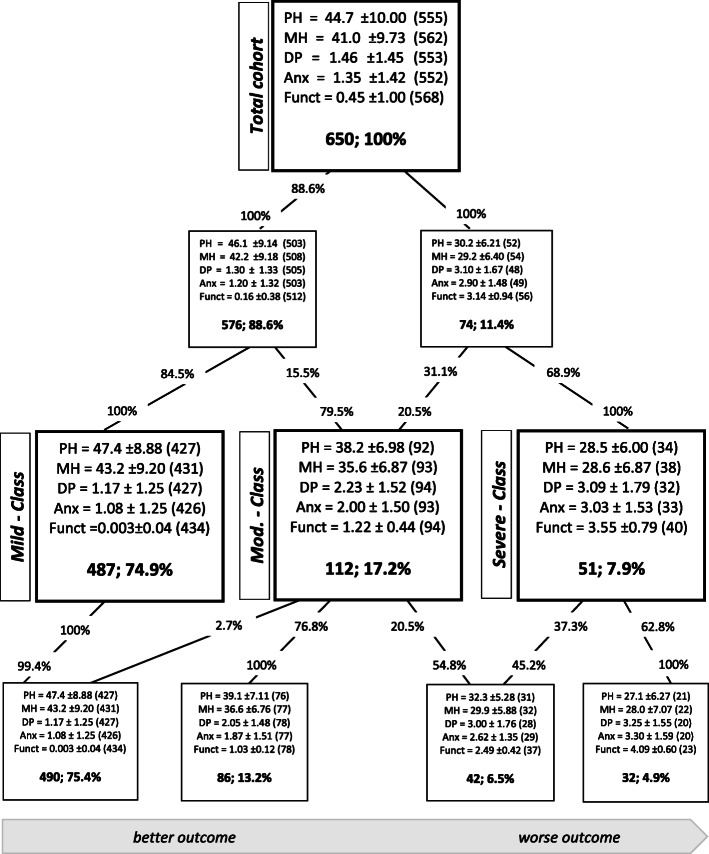
Class Evolution Tree of the four empirically most strongly supported models from the latent profile analysis. The bold numbers indicate absolute and relative frequency. The depicted variables are patient-reported outcomes. Abbreviations: PH = physical health; MH = mental health; Dep = depressive symptoms; Anx = anxiety; Funct = functional status; mod. = moderate

According to the information criteria, models with freely estimated within-class correlations between variables and freely estimated within-class variances showed the best fit to the data (Supplemental Table I). Among them, only models with one to four classes had at least 30 participants in the smallest class. Of these four models, information criteria supported the four-class model, while the likelihood ratio tests supported the two-class solution. Therefore, we investigated the models with two, three, and four classes further in the class evolution tree analysis, which suggested a successive differentiation according to the severity of impairment as assessed by patient-reported outcome measures (Fig. 1). In summary, the latent profile analysis suggested a roughly unidimensional classification scheme with the severity of impairment of one’s health being largely independent of the specific health domains and allowing for a differentiation between two, three, or four grades of impairment.

**Fig. 2:**
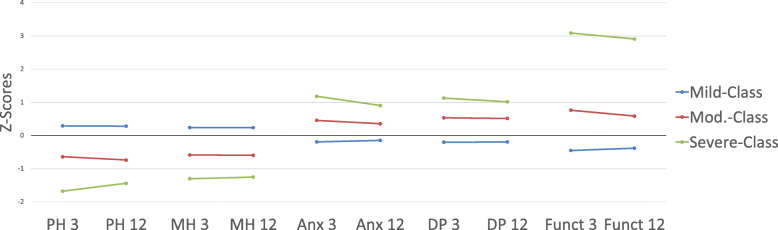
Patient-reported outcomes after 3 and 12 months clustered in three classes of mild (1), moderate (2), and severe (3) impairment. Physical health (PH) and mental health (MH) were assessed by PROMIS-10, anxiety (Anx) and depressive symptoms (DP) by the PHQ-4, dependencies in functional status (Funct) concerning dressing, toileting and walking by the ICHOM-SSS after 3 and 12 months of diagnosis. Outcomes were clustered by Latent Profile Analysis in three classes (1 = blue, 2 = red, 3 = green). The different values of the items were related to the Z-Score for comparability. Abbreviations: PH = physical health; MH = mental health; Anx = anxiety; DP = depressive symptoms; PROMIS-10 = Patient-reported Outcomes Measurement Information System Short Form-10; PHQ-4 = Patient Health Questionnaire-4; ICHOM-SSS=International Consortium for Health Outcome Measurement Standard Set for Stroke

In the two-class model, 576 (88.6%) patients showed milder and 74 (11.4%) more severe impairment. In the three-class model, 487 (74.9%) patients reported of mild, 112 (17.2%) of moderate, and 51 (7.8%) of severe deficits (Fig. 2). Division in four classes led to 490 (75.4%) patients, who reported of mildly impaired, 86 (13.2%) of moderately, 42 (6.5%) of moderately to severely, and 32 (4.9%) of severely impaired health. For further investigation, we focused on the three-class model, because it offers a sufficiently differentiated but still practical classification with divisible severity and class size. Patients’ characteristics in the three-class model are displayed in Table 1.

**Table 1:** Distribution of patients’ characteristics in the three-class model

	Mild-Class *N* = 487	Moderate-Class *N* = 112	Severe-Class *N* = 51
Variable			
Age, mean (SD)	70.84 (12.76)	74.62 (12.02)	78.61 (10.48)
Gender			
Female	224 (46.0%)	53 (47.3%)	33 (64.7%)
Male	263 (54.0%)	59 (52.7%)	18 (35.3%)
Living situation: alone	174 (35.7%)	38 (33.9%)	26 (56.5%)
Living location (at 90d)			
At home without external support	381 (88.2%)	54 (57.4%)	3 (7.7%)
Stroke type			
Acute ischemic stroke	323 (66.3%)	91 (81.3%)	41 (80.4%)
Intracranial hemorrhage	22 (4.5%)	9 (8.0%)	9 (17.6%)
Transient ischemic stroke	142 (29.2%)	12 (10.7%)	1 (2.0%)
Lesion area			
Hemisphere left/right (%)	222/163	52/42	23/23
Brainstem	89 (18.3%)	16 (14.3%)	3 (5.9%)
Both hemispheres or brainstem+	12 (2.5%)	2 (1.9%)	2 (3.9%)
MRI at initial inpatient stay	188 (38.6%)	43 (38.4%)	12 (23.5%)
Stroke severity [NIHSS] median (IQR)	1 (0;3.5)	4 (2;9)	14 (6;16)
No stroke symptoms [0]	182 (37.4%)	10 (8.9%)	0 (0.0%)
Mild stroke symptoms [1–4]	212 (43.5%)	49 (43.8%)	11 (21.6%)
Mod. stroke symptoms [5–15]	81 (16.6%)	48 (42.9%)	25 (49.0%)
Mod. to sev. Stroke symptoms [16–21]	8 (1.6%)	4 (3.6%)	10 (19.6%)
Severe stroke symptoms [21–42]	4 (.8%)	1 (.9%)	5 (9.8%)
Stroke treatment			
Thrombolysis	88 (18.1%)	25 (22.3%)	13 (25.5%)
Endovascular thrombectomy	49 (14.9%)	16 (17.4%)	19 (46.3%)
Cardio-vascular risk factors			
Prior ischemic stroke	81 (16.6%)	22 (19.8%)	10 (20.0%)
Prior transient ischemic stroke	18 (3.7%)	6 (5.4%)	0 (0.0%)
Prior myocardial infarction	32 (6.6%)	14 (12.5%)	1 (2.0%)
Coronary artery disease	57 (11.8%)	7 (6.3%)	6 (12.0%)
Atrial fibrillation	71 (14.6%)	35 (31.5%)	17 (34.0%)
Diabetes mellitus	62 (12.8%)	27 (24.3%)	11 (22.0%)
Hypertension	290 (59.9%)	75 (67.6%)	37 (74.0%)
Hyperlipidemia	67 (14.2%)	14 (12.8%)	3 (6.3%)
Smoking	100 (21.8%)	18 (18.6%)	4 (9.8%)
Length of stay, mean (SD)	5.74 (4.60)	8.92 (6.45)	11.12 (9.73)
Discharge destination: Back home	381 (78.6%)	42 (37.5%)	5 (9.8%)
Stroke recurrence within 12 months	66 (13.6%)	21 (18.8%)	11 (21.6%)

*Abbreviations*: *N* number, *SD* Standard deviation, *NIHSS* National Institute of Health Stroke Scale

### Characteristics associated with class membership

Assessed in univariate comparison of one class with one other (Supplemental Table II), the average age continuously increased from 71 years in the mildly affected class one to 75 years in the moderately affected class two (*p* = 0.005; OR = 1.03), and from class two further to 79 years in the severely affected class three (*p* = 0.046; OR = 1.03). The severity of initial neurological deficits assessed by the NIHSS paralleled worse PROs with a median of 4 (IQR:2,9) in the class of moderately affected PROs compared to a median of 1 (IQR:0,3.5) in the mildly affected class (*p* < 0.001; OR = 2.30), and a median of 14 (IQR:6,16) in the most severely opposed to 4 (IQR:2,9) in the moderately affected class (*p* < 0.001; OR = 3.19). Length of initial inpatient stay showed an increase from mildly to the moderately affected class (*p* < 0.001; OR = 1.10). Stroke subtype differed between these classes as well (*p* < 0.001), as did cardiovascular risk factors: In the class of mild impairment, 13% had diabetes compared to 24% in the class of moderate impairment (*p* = 0.002; OR = 2.19), the rate of atrial fibrillation was 15% compared to 32% (*p* < 0.001; OR = 2.70), and 7% had prior MI compared to 13% (*p* = 0.037; OR = 2.03). The class of moderate and severe impairment differed in the percentage of patients living alone (*p* = 0.010; OR = 2.53), a higher rate of endovascular thrombectomy (*p* < 0.001; OR = 1.85) and women being more frequent in the severely than the moderately affected class (*p* = 0.041; OR = 2.04).

The multivariate model of 595 patients belonging to the class of mild or moderate impairment in PROs showed that initial severity of neurological deficits (*p* < 0.001; OR = 1.74), length of initial inpatient stay (*p* < 0.001; OR = 1.08), atrial fibrillation (*p* = 0.004; OR = 2.20), and diabetes (*p* = 0.021; OR = 1.91) predicted membership to the moderately affected class (Table 2).

**Table 2:** Factors predicting membership of the moderately compared to mildly affected class

*N* = 595	OR (CI)	*P*-value
Age	1.02 (1.00, 1.04)	0.058
Stroke subtype	0.83 (0.60, 1.16)	0.280
Initial NIHSS	1.74 (1.29, 2.35)	< 0.001
Length of initial inpatient stay	1.08 (1.04, 1.12)	< 0.001
Prior myocardial infarction	1.52 (1.01, 3.17)	0.262
Atrial fibrillation	2.20 (1.30, 3.75)	0.004
Diabetes	1.91 (1.10, 3.30)	0.021

Comparing membership of the class of moderate with the class of severe impairment in a model of 158 patients, only initial severity of neurological symptoms (*p* < 0.001; OR = 3.51) was significantly associated with membership to class three (Table 3).

**Table 3:** Factors predicting membership of the severely compared to moderately affected class

*N* = 158	OR (CI)	*P*-value
Age	1.04 (0.99, 1.08)	0.117
Sex = Female	1.75 (0.68, 4.52)	0.247
Initial NIHSS	3.51 (1.76, 6.99)	< 0.001
Living alone	1.52 (0.61, 3.79)	0.371
Endovascular thrombectomy	1.85 (0.70, 4.94)	0.216

## Discussion

In our study of consecutive stroke patients treated in clinical practice, grading of patients by classes of PROMs up to 12 months after stroke identified different profiles of patients’ demographic and clinical characteristics. Class membership was associated with stroke severity, stroke subtype, pre-stroke living situation, and cardio-vascular comorbidities to a different degree. In a three-class-model, the severity of initial neurological deficits was the only predictor of class membership across all three classes from patients with only minor impairment to those with severely impaired health status. In addition, history of diabetes or atrial fibrillation, and length of in-hospital stay predicted assignment to the class of moderately impaired health status instead of the class of mild impairment.

The cohort studied here consists of unselected patients from a university stroke center admitted as inpatients and followed prospectively after acute treatment. With a mean of 75 years, high frequency of cardiovascular risk factors, and the observed distribution of stroke subtypes, our cohort is representative for the general stroke population [10, 26]. In this sense, our study adds new data to class analysis of stroke outcome based on PROMs, as the only published comparable was a retrospective analysis comprised of younger patients with overall less severe impairment [15]. We used the ICHOM-SSS with its standardized selection of PROMs to assess its value in routine care and enable comparability. The parameters recorded with the ICHOM-SSS and PHQ-4 provided sufficient information to calculate class models with a plausible distribution and reflection of patient characteristics as compared to the previous class analysis after stroke [15]. The frequency of patients with scores indicative for anxiety and depressive symptoms matches previous analyses as well [4]. In our study, absolute values of PROMs and class differences remained rather stable between 3 and 12 months after stroke. This observation stands in contrast to previously reported relevant improvements of externally rated outcomes using the modified Ranking Scale or Barthel index in the same time-frame and might indicate the different scope of PROMs as compared to assessments by health professionals [9, 17].

The differences of each patient class to one another in severity of neurological deficits in our models are similar to the ones described in the retrospective analysis of an outpatient setting, and the association of worse values of PROMs after stroke with greater initial neurological deficits has been described before [1, 33]. Beyond initial stroke severity, history of diabetes and atrial fibrillation predicted assignment to the moderately affected instead of the mildly affected class. This indicates that treatable cardiovascular comorbidities are especially relevant for self-reported outcomes of patients with mild to moderate initial neurological deficits. Negative self-affirmation in controlling their comorbidity following the stroke event might in the mild to moderately affected groups not be outweighed by severe neurological deficits and the reason for the influence of comorbidity on PROMs [2, 13]. These findings might thus point to new treatment targets after stroke as psychotherapeutic support in handling of comorbidities. In support of this judgement, the Northern-Manhattan-Study found that diabetes predicts decline in self-assessed Barthel index [6].

Length of initial inpatient stay independently predicted membership of the moderately affected class opposed to the mildly. This is in line with data showing that patients with moderate neurological deficits benefit most from early supportive discharge [3, 19]. The clearer characterization of the moderate outcome class may help in identifying patients that could benefit from early supportive discharge beyond initial neurological deficits, like patients with the mentioned cardiovascular comorbidities. These findings demonstrate a possible clinical value of class-analyses. In case of more severely affected patients the best time point for discharge remains unclear and is supposedly influenced by multiple substantial medical reasons, such as complications of therapy [22].

The analysis of parameters distinguishing the classes of patients with moderately and severely decreased self-reported outcomes was limited by the smaller sample sizes with 122 and 51 patients assigned to these classes. Severity of neurological symptoms was identified as the only significant parameter differentiating between these two classes. However, living alone predicted assignment to the class with severely impaired health status in univariate analysis, and lack of significance after adjustment for cofactors might be related to sample size. The impact of living status on initial stroke severity, e.g., by delayed presentation of patients living alone, has been described previously [25], and the potential effect on further course of disease is discussed controversially [8, 12, 32]. Our data point towards a possible association of living alone with worse self-reported health status in moderately to severely affected stroke patients, but need to be verified in a larger cohort. The higher rate of endovascular treatment in the class of severe impairment is supposedly a cause of more severe stroke symptoms and large vessel occlusions in this group [29]. Though women were more frequent in the severely impaired class, sex did not significantly contribute to distinguish it from the class of moderately affected patients and we cannot confirm its negative influence on PROs described for mild to moderately affected outpatient stroke patients in our inpatient stroke center cohort [15].

One limitation of our study is the reduced generalizability due to the single-center design representing patients from a university stroke center located in an urban area. The large number of patients lost to follow-up represents a further limitation. To assess the potential influence of missing follow-up data on the results, we performed a sensitivity analysis based on multiple imputation in a previous analysis. This analysis of the imputed full dataset revealed no substantial differences in outcome and predictive factors [27]. Another limitation concerns the comparison between the moderate and severe class, which might underestimate associations due to small class size of patients with severe impairment.

## Conclusions

Altogether, our study demonstrates the possibility and potential clinical benefit of classifying patients in the course of acute stroke treatment by a standard set of PROMs for risk stratification. The differentiated view on patients separated by classes reveals differences in characteristics noticeable only in subgroups of outcome, relevant as potential driving factors of worse long-time outcome, and thus also as potential targets for new treatment approaches. The rarity of distinguishing characteristics shows the limits of a standard set. Class models from focused PROMs may represent the next step towards a more individually tailored treatment within the framework of value-based medicine.

## Supplementary Information

**Additional file 1.**

## Data Availability

Deidentified individual participant data will be made available upon reasonable request and where necessary after approval of the ethics committee by the corresponding author within 24 months after publication. Additional information can be taken from our study protocol.

## Abbreviations

PROMs: Patient reported outcome measures
PROMIS: Patient-reported Outcomes Measurement Information System
PROMIS-10: PROMIS 10-Question Short Form
ICHOM-SSS: International Consortium for Health Outcome Measurement Standard Set for Stroke
PHQ-4: Patient Health Questionnaire-4
PH: Physical health
MH: Mental health
Anx: Anxiety
DP: Depressive symptoms
AIS: Acute ischemic stroke
TIA: Transient ischemic attack
ICH: Intracerebral hemorrhage
NIHSS: National Institutes of Health Stroke Scale
ABIC: Adjusted Bayesian Information Criterion
VLMR-LRT: Vuong–Lo–Mendell–Rubin likelihood ratio test
LMR-ALRT: Lo–Mendell–Rubin adjusted likelihood ratio test
